# SWUAV-DANet: A Severe-Weather UAV Dataset and Dynamic AlignAir Network for Robust Aerial Vehicle Detection

**DOI:** 10.3390/s26092793

**Published:** 2026-04-30

**Authors:** Longze Zhang, Yihong Li

**Affiliations:** School of Cyberspace Security (School of Cryptology), Hainan University, 58 Renmin Avenue, Haikou 570228, China; chien_abyss@hainanu.edu.cn

**Keywords:** UAV, severe weather, aerial vehicle detection, adverse-weather object detection, cross-scale alignment, deformable detection head, real-time detection

## Abstract

Unmanned aerial vehicle (UAV) aerial object detection is increasingly important for traffic monitoring, emergency rescue, and environmental perception. However, vehicle detection in heavy rain, dense fog, blizzards, and backlit night scenes suffers from target information loss, feature misalignment, and unstable performance. We, therefore, construct a new severe-weather UAV dataset, Severe-Weather UAV (SWUAV), and propose the real-time Dynamic AlignAir Network (DANet). SWUAV contains 18,195 red–green–blue (RGB) aerial images covering 12 adverse weather/illumination conditions with 236,392 vehicle instances. After the high-resolution backbone features, we insert a cross-scale adaptive alignment module that performs adaptive channel calibration, contrastive self-attention, and geometric/semantic remapping to reduce scale drift/mismatch, suppress noise, and strengthen degraded target cues; we then design a dynamic adaptive alignment head (DAAH) with a shared encoder and a deformable regression branch to mitigate classification–regression mismatch under adverse conditions while further reducing complexity. On SWUAV, DANet raises the YOLOv11-s baseline average precision (AP)/AP50 (AP at intersection over union, IoU = 0.50) from 43.9%/62.6% to 46.9%/64.8%, with only 8.65 M parameters, 22.7 giga floating-point operations (GFLOPs), and a 323.47 frames-per-second (FPS) end-to-end throughput (3.09 ms per image at batch size 16), outperforming EdgeYOLO-s and RT-DETR. The dataset and code are publicly available.

## 1. Introduction

Deep neural network detectors have progressed rapidly on natural images [1,2] and been extended to aerial imagery. Directly transferring generic models to low-altitude unmanned aerial vehicle (UAV) scenes remains difficult [3]: (1) rain/snow streaks, haze scattering, backlighting, and night backgrounds significantly weaken target visibility and edges, causing feature and model mismatch; (2) degradation mechanisms are heterogeneous in severe weather: atmospheric scattering mainly explains fog/haze attenuation and airlight effects, while rain/snow streak occlusion, accumulation effects, and nighttime/backlit glare introduce additional non-uniform disturbances, leading to marked performance drops in out-of-distribution scenes, such as blizzards, dense fog, heavy rain, and night [4,5,6,7].

Public UAV datasets have spurred research but share gaps: VisDrone-DET 2019 [8] focuses on urban flights with few extreme-weather or night samples. UAVDT [9] stresses traffic monitoring, yet many images come from consecutive video frames with high redundancy. UAV123 [10] targets single-object tracking with one class. DroneVehicle [11] offers fine-grained vehicle splits but mainly in clear daytime. Overall, blizzards, dense fog, heavy rain, backlight, and night are under-represented, and dense multi-vehicle scenes are limited, making robustness and cross-dataset generalization hard to evaluate; large remote-sensing sets (e.g., DOTA, xView) likewise lack low-altitude severe-weather coverage.

We, therefore, build the Severe-Weather UAV (SWUAV) dataset: 18,195 red–green–blue (RGB) aerial images covering blizzards, freezing rain, dense fog, heavy rain, and backlit night across 12 weather/illumination combinations, with diverse traffic scenes. It is intended for multi-scale detection under adverse conditions and for models that must adapt to complex environmental interference.

Detection models fall into two-stage and one-stage paradigms [12,13,14,15,16,17]. Two stages (e.g., Faster R-CNN) offer higher accuracy but heavier models; one stage (YOLO, SSD, RetinaNet) balances accuracy and speed. Detection Transformer (DETR)-style transformers unify proposal generation and post-processing but demand more computing and training stability. In severe-weather UAV scenes with small and crowded objects, limitations persist: (1) shallow spatial details are diluted, causing cross-scale semantic–spatial mismatch and missed or drifted detections; (2) heads across scales and between classification/regression lack alignment and are noise-sensitive under low visibility; and (3) a redundant structure and large parameters hinder lightweight edge deployment. Recent adverse-weather detection studies further explore image-adaptive preprocessing (IA-YOLO [18], ERUP-YOLO [19]), restoration-detection coupling (TogetherNet [20], DTFA [21]), feature decorrelation (ILM/DAM [22]), and lightweight multiscale redesigns (MASFNet [23], YOLO-FOD [24]). These methods report gains on adverse-weather road-scene benchmarks, but most focus on ground-view domains and do not explicitly address the coupled cross-scale and task-alignment issues in low-altitude UAV traffic scenes.

To address this, we propose the Dynamic AlignAir Network for complex-weather UAV vehicle detection: the Cross-scale Adaptive Contrastive Tokenizer (CACT) aligns shallow geometry with deep semantics in high-resolution space, and the Dynamic Adaptive Alignment Head (DAAH) aligns classification and regression across scales on shared encodings, improving accuracy and robustness under adverse-weather conditions while retaining real-time performance.

## 2. Related Work

Unlike ground traffic monitoring or natural-scene detection, UAV detection under adverse weather faces unique perception obstacles: low-altitude platforms must maintain stable recognition amid continuous rain, snow reflections, haze scattering, and backlit nights, while the aerial viewpoint amplifies occlusion, scale changes, and target density. For fog/haze-dominated visibility degradation, we adopt the classical atmospheric-scattering approximation [4,5]:

(1) $$I_{c}\left(x\right)=J_{c}\left(x\right)\;t_{c}\left(x\right)+A_{c}\left(1-t_{c}\left(x\right)\right)+R_{c}\left(x\right)+N_{c}\left(x\right),$$

(2) $$t_{c}\left(x\right)=\exp\left(-\beta_{c}d\left(x\right)\right).$$

Here $I_{c}$ is the camera observation, $J_{c}$ is the true radiance, $t_{c}$ is the transmission along the line of sight, and $A_{c}$ is the background light. In fog/haze conditions, a larger $\beta_{c}$ and a stronger $A_{c}$ reduce contrast, blur edges, and shift colors. For rain/snow and nighttime/backlit scenes, additional effects (e.g., dynamic streak occlusion, accumulation, and non-uniform glare) are not explicitly modeled by Equations (1) and (2) and are represented phenomenologically by $R_{c}$ and $N_{c}$. This formulation serves as a compact mixed-degradation abstraction for performance analysis in low-visibility UAV detection.

Existing public UAV datasets—VisDrone-DET 2019 [8], UAVDT [9], UAV123 [10], and DroneVehicle [11]—mainly cover clear daytime or mild weather, with limited blizzard, dense fog, heavy rain, backlight, or night samples. Many frames are highly repetitive, categories can be single, and high-density traffic scenes are scarce, making it hard to assess robustness and generalization. Large remote-sensing sets (e.g., DOTA, xView), likewise, lack low-altitude severe-weather support. SWUAV helps narrow these gaps by providing 18,195 images over 12 weather/illumination conditions, 30 traffic scenes, and 236,392 vehicle annotations (car, truck, bus, van, freight car), improving extreme-weather coverage, scene diversity, and dense multi-class detection.

General object detection has evolved from two-stage to one-stage to Transformer frameworks [1,2]. For UAV imagery (large view, small and dense targets, pose changes, computing limits), specialized models such as Efficient-YOLO [25], BPD-YOLO [26], ELNet [27], and RSW-YOLO [28] enhance lightweight UAV detection under small-and-dense settings.

In adverse-weather detection, IA-YOLO [18] and ERUP-YOLO [19] introduce differentiable image-adaptive preprocessing before detection, TogetherNet [20] and DTFA [21] bridge restoration and detection through joint or teacher-guided feature alignment, independence-based decorrelation learning [22] reduces spurious weather-feature correlations at instance and image levels, and lightweight architectures such as MASFNet [23] and YOLO-FOD [24] strengthen multiscale fusion under computing constraints. These advances improve robustness on adverse-weather road-scene benchmarks, but severe-weather low-altitude UAV settings remain underexplored.

For severe-weather UAV detection, three gaps remain: limited benchmark coverage for dense low-altitude adverse-weather traffic, the insufficient modeling of UAV-specific cross-scale semantic–spatial mismatch, and weak joint alignment of classification/regression across scales. We thus propose DANet (Dynamic AlignAir Network) to improve accuracy and stability in severe-weather UAV detection.

Our contributions are summarized as follows:

- SWUAV dataset. To the best of our knowledge, we release one of the few UAV traffic detection datasets explicitly designed for severe-weather and low-visibility scenarios, with 236k vehicle instances across blizzard, dense fog, heavy rain, backlight, and night scenes in 30 traffic settings, supporting robustness and domain-generalization research.
- Dynamic AlignAir Network. CACT plus DAAH provide an effective real-time solution for traffic detection under extreme weather and complex environments.
- Comprehensive evaluation. Extensive experiments benchmark current real-time detectors and validate SWUAV and DANet; the data and code are publicly available for follow-up work.

## 3. Severe-Weather UAV Dataset

**Figure 1:**
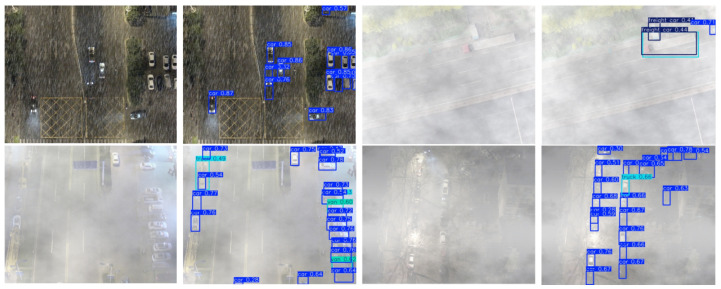
Example detection results for specific object categories in the Severe-Weather UAV (SWUAV) dataset.

To advance UAV traffic detection under complex weather, we build the SWUAV dataset (examples in Figure 1).

**Figure 2:**
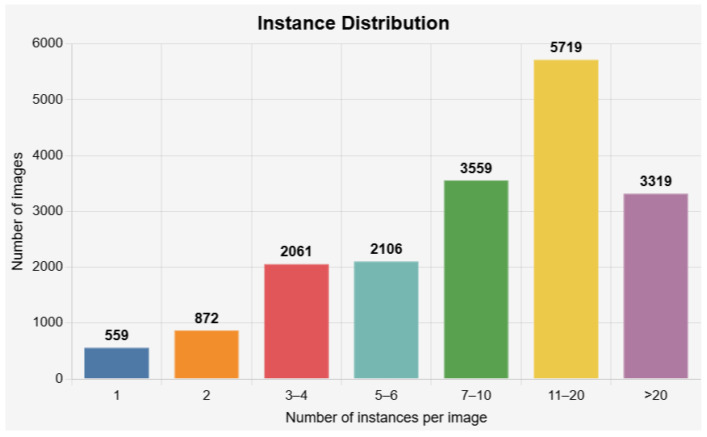
Distribution of the number of instances per image in the SWUAV dataset.

It contains 18,195 RGB aerial images at 640×512 resolution, covering blizzards, dense fog, heavy rain, backlit night, and other adverse scenes, with 236,392 annotated vehicle instances (car, truck, bus, van, freight car). Each image shows about 13 targets on average (distribution in Figure 2), spanning smooth road traffic, as well as dense toll plazas, river-crossing interchanges, port yards, and logistics hubs. Below, we describe the construction process and statistics of SWUAV.

### 3.1. Dataset Collection, Annotation, and Splitting

Dataset creation followed three phases: collection/filtering, annotation, and splitting. Data were collected from numerous traffic sites across multiple urban regions using four UAV units from the same DJI-series consumer platform family and were supplemented with publicly available aerial footage captured under severe-weather conditions. The collected videos cover diverse traffic environments, viewpoints, and weather/illumination conditions. Candidate frames were manually screened to remove near-duplicates and low-quality samples, ensuring annotation quality and visual consistency. Although camera parameters vary slightly across units, this variability reflects realistic UAV imaging conditions, rather than controlled laboratory settings. To mitigate potential geographic or scene bias, both the training and testing splits include samples collected from multiple regions. Annotation followed an “auto-suggest + manual refinement” workflow: a vision foundation model generated candidate boxes, annotators corrected and completed them in CVAT, and class consistency was checked in a unified review. YOLO-format instance boxes ensure compatibility; the totals are 207,427 cars, 10,451 trucks, 6144 buses, 5914 vans, and 6456 freight cars. For splitting, SWUAV uses roughly 7:1:2: 12,680 train, 1947 val, 3568 test images with class ratios aligned, forming a moderately difficult benchmark for severe-weather UAV detection/segmentation and for reproducible baselines.

### 3.2. Dataset Characteristics and Statistics

**Figure 3:**
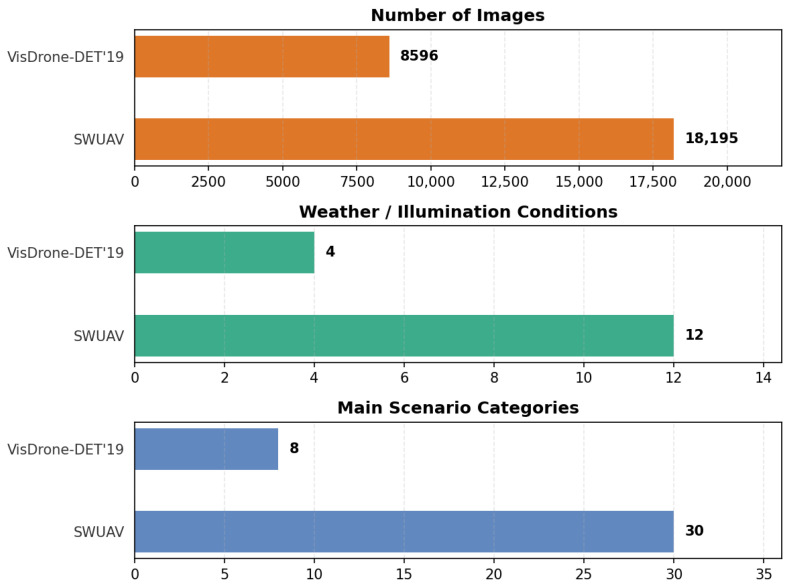
Comparison of SWUAV and VisDrone-DET 2019 datasets.

Existing UAV detection datasets like VisDrone-DET 2019 mainly cover clear or overcast scenes. UAVDT focuses on city traffic with fair weather. UAV123/DroneVehicle target tracking or daytime vehicle identification. Samples for extreme weather and night illumination remain scarce, weakening robustness under low visibility and complex lighting. To address this gap, SWUAV spans 12 real weather/illumination combinations (Table 1) and significantly expands image scale, weather diversity, and scene complexity compared with VisDrone-DET 2019 (Figure 3).

Instance counts and categories. SWUAV provides 236,392 vehicle instances across five categories (car, truck, bus, van, freight car), richer than UAVDT and DroneVehicle. Over 70% of scenes contain more than seven instances; 31.43% fall in the 11–20 range, and 18.24% exceed 20 (Figure 2), far denser than VisDrone-DET or UAV123, enabling stress tests of multi-target discrimination and robustness in congested traffic.

**Figure 4:**
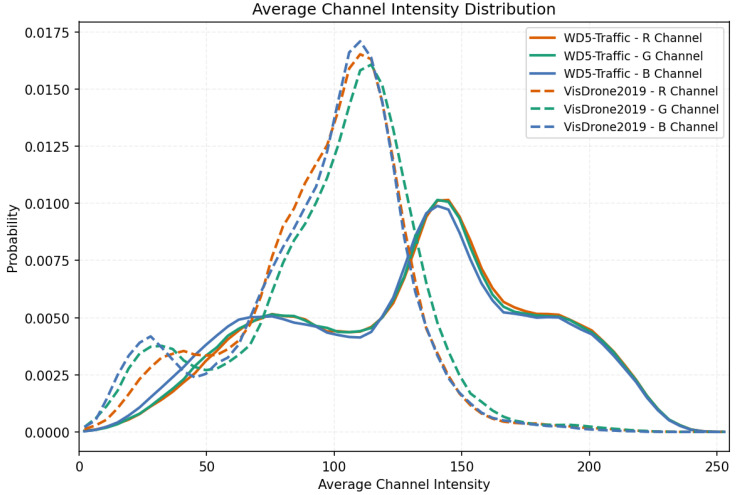
SWUAV average channel intensity distribution.

Channel-intensity characteristics. We compute the mean intensity per channel across all images and compare with VisDrone2019 (mainly clear daytime) in Figure 4. VisDrone peaks sharply at 100–115, indicating mid-level brightness typical of clear daylight. SWUAV shows broader dual peaks with a high-intensity mode at 140–160 but lower overall peak height and flatter curves, with larger variance. This reflects coexisting low-light/cool regions (fog, rain, snow) and high-brightness regions (backlight, snow reflections, nighttime lamps), giving a wider luminance range and combined color attenuation. SWUAV, therefore, covers more complex illumination and weather, helping models learn robust features under low visibility, strong contrast, and color distortion.

**Figure 5:**
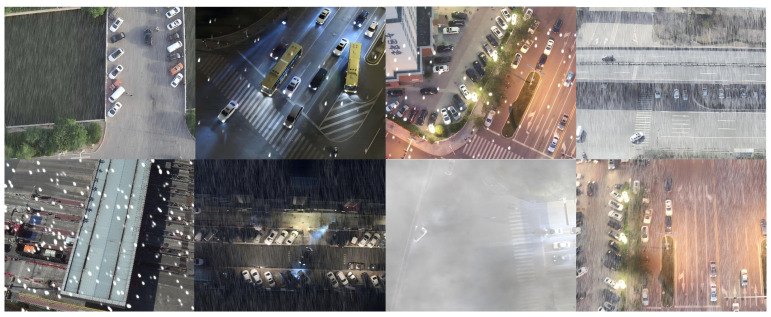
Examples of scene diversity in the SWUAV dataset.

Scene diversity. SWUAV uses unified imaging settings for real-time deployment while covering 30+ scenes, such as toll plazas, river-crossing interchanges, port yards, highway ramps, and residential parking lanes (Figure 5), capturing disturbances like wet lenses, beacon glare, condensation, and reflections. Compared with UAV123 and DroneVehicle (mainly clear daytime) and VisDrone-DET (clear/overcast with few night scenes), SWUAV offers high-density, multi-class vehicle distributions under extreme weather, low light, and complex backgrounds, enabling challenging yet practical evaluation and design of feature enhancement/alignment strategies for severe weather.

Quantitative weather distribution comparison: Table 2 summarizes per-condition image counts for SWUAV and the available weather labels in UAVDT-M. Note that UAVDT only provides daylight/night/fog attributes, so other severe-weather categories are not annotated.

## 4. DANet Network Model

**Figure 6:**
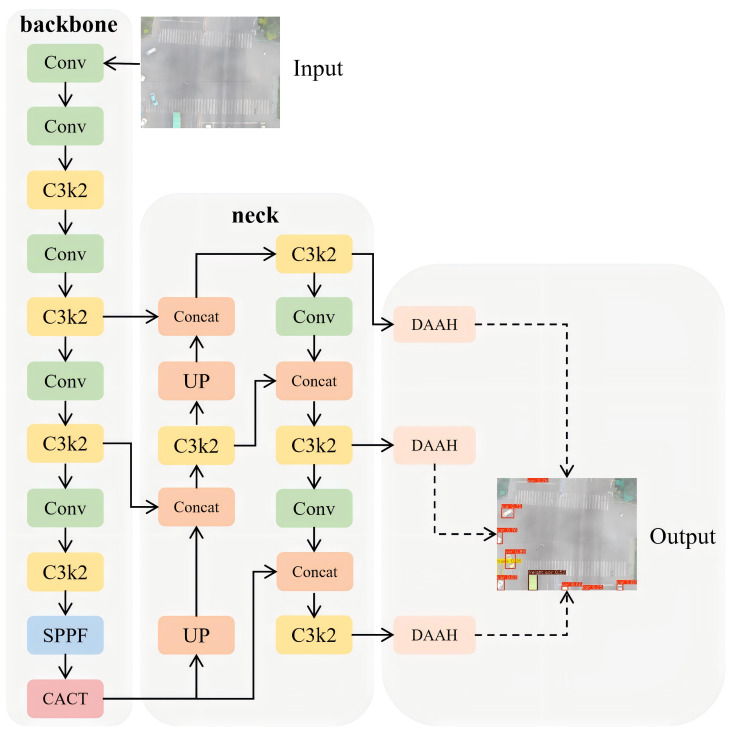
Overall framework of the Dynamic AlignAir Network.

Figure 6 shows the overall DANet architecture for UAV vehicle detection under complex weather. It introduces two core modules: the Cross-scale Adaptive Contrastive Tokenizer (CACT) and the Dynamic Adaptive Alignment Head (DAAH). CACT uses cross-scale contrastive attention in a loop of channel splitting, dynamic calibration, complementary mapping, and feature aggregation to align shallow geometry with deep semantics at a high resolution, improving separability and stability for hard or confusing targets. DAAH applies scale-adaptive calibration on shared encodings, using deformable convolutions for geometric alignment in regression and probabilistic gating for semantic focus in classification, yielding consistent multi-scale predictions and improved output stability under adverse-weather conditions.

### 4.1. Cross-Scale Adaptive Contrastive Tokenizer (CACT)

**Figure 7:**
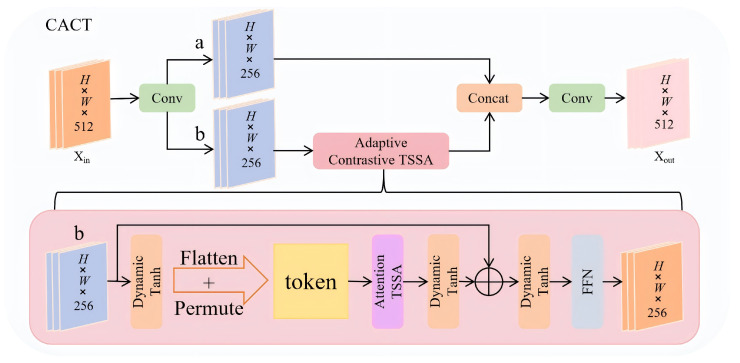
Framework of the Cross-Scale Adaptive Contrastive Tokenizer and Token-Shift Self-Attention.

Repeated down-sampling in the backbone weakens spatial details and breaks semantic consistency across scales, causing the detection head to make inconsistent decisions on the same target. We propose the Cross-scale Adaptive Contrastive Tokenizer (CACT) to address this. Using the SPPF output P5 feature

(3) $$\begin{array}{cc} X_{\mathrm{in}} & \in R^{B\times 512\times H\times W} \end{array}$$

as input, CACT performs channel splitting, stacked Adaptive Contrastive TSSA (ACT) blocks, and channel reconstruction to encode shallow geometry as dynamic tokens and realign them in a high-resolution semantic space. The structure is shown in Figure 7.

#### 4.1.1. Channel Split

We first apply cv1 (Conv 1×1 + BN + SiLU) for linear rearrangement while preserving the spatial size and activating channels. The 512 channels are then divided into two equal branches: ${\mathrm{branch}}_{a}$ keeps the original semantics as a long-range residual, and ${\mathrm{branch}}_{b}$ enters the ACT path for explicit calibration:

(4) $$\begin{array}{cc} \left[{\mathrm{branch}}_{a},{\mathrm{branch}}_{b}\right] & =\mathrm{Split}(\mathrm{cv}1\left(X_{\mathrm{in}}\right)),\\ {\mathrm{branch}}_{a},{\mathrm{branch}}_{b} & \in R^{B\times 256\times H\times W}. \end{array}$$

This preserves shallow spatial cues for later enhancement, providing an alignment space for adaptive attention.

#### 4.1.2. Adaptive Contrastive TSSA

The Adaptive Contrastive Token-Shift Self-Attention (ACT) block is the key component of CACT, aiming to recalibrate multi-scale semantics with minimal computing (Figure 7). On input ${\mathrm{branch}}_{b}\in R^{B\times 256\times H\times W}$, it performs dynamic modulation, sequence modeling, contrastive attention, second-stage modulation, and channel aggregation, with each step conditioning the next to form complementary mappings.

#### 4.1.3. Channel-Wise Adaptive Tuning (DynamicTanh 1)

Feeding raw features directly into attention would amplify background noise and amplitude drift. We first apply DynamicTanh modulation to suppress noise and stabilize amplitudes:

(5) $$\begin{array}{cc} X & ={\mathrm{DYT}}_{1}\left({\mathrm{branch}}_{b}\right), \end{array}$$

(6) $$\begin{array}{cc} X_{b,c,u,v} & =\gamma_{1,c}\tanh\;\left(\alpha_{1}\cdot {\left({\mathrm{branch}}_{b}\right)}_{b,c,u,v}\right)+\beta_{1,c}, \end{array}$$

where $\alpha_{1}\in R$ and $\gamma_{1},\beta_{1}\in R^{C}$.

#### 4.1.4. Explicit Sequencing for Long-Range Dependencies (Flatten and Permute)

Convolutional features are local; to expose long-range relations, we flatten the feature map into a token sequence:

(7) $$\begin{array}{cc} T & =\mathrm{reshape}\left(X\right)\in R^{B\times N\times C},\\ N & =H\times W,\;C=256. \end{array}$$

Sequencing provides an attention-friendly representation so self-attention can model distant semantic coupling beyond convolutional receptive fields.

#### 4.1.5. Contrastive Multi-Head Self-Attention (Attention TSSA)

To avoid ambiguous notation, we provide the implementation-equivalent form of Attention TSSA. In ACT, attention is computed from token statistics through head-wise saliency gating and contrastive shrinkage:

(8) $$\begin{array}{cc} W & =\mathrm{reshape}\left(TW_{\mathrm{in}}\right)\in R^{B\times h\times N\times d},\;C=hd,\\ {\hat{W}}_{b,h,n,j} & =\frac{W_{b,h,n,j}}{\sqrt{\sum_{n^{\prime }=1}^{N}W_{b,h,n^{\prime },j}^{2}+\varepsilon }},\\ E_{b,h,n} & =\tau_{h}\sum_{j=1}^{d}{\hat{W}}_{b,h,n,j}^{2},\\ \Pi_{b,h,n} & ={\left[{\mathrm{Softmax}}_{h}\left(E_{b,:,n}\right)\right]}_{h}=\frac{\exp(E_{b,h,n})}{\sum_{h^{\prime }=1}^{h}\exp\left(E_{b,h^{\prime },n}\right)},\\ {\bar{\Pi }}_{b,h,n} & =\frac{\Pi_{b,h,n}}{\sum_{n^{\prime }=1}^{N}\Pi_{b,h,n^{\prime }}+\varepsilon },\\ \psi_{b,h,j} & =\sum_{n=1}^{N}{\bar{\Pi }}_{b,h,n}\;W_{b,h,n,j}^{2},\\ {\mathrm{Attn}}_{b,h,j} & =\frac{1}{1+\psi_{b,h,j}},\\ Y_{b,h,n,j} & =-W_{b,h,n,j}\;\Pi_{b,h,n}\;{\mathrm{Attn}}_{b,h,j},\\ T^{\prime } & =\mathrm{merge}\left(Y\right)\;W_{\mathrm{out}}. \end{array}$$

Here, Π is normalized across heads for each token (head-wise competition), while $\bar{\Pi }$ is further normalized across tokens within each head. The contrast function is defined as contrast(T)≜ψ, where ψ is the weighted channel energy. Therefore, Attn=1/(1+ψ) performs inverse-energy shrinkage, suppressing over-activated channels.

#### 4.1.6. Complexity Analysis of ACT

The ACT attention core above is implemented via normalization, reduction, and weighted aggregation over tokens, with complexity O(BhNd) and no explicit N×N affinity construction. Considering the full ACT block, the major terms are

(9) $$O\left(BNC^{2}\right)\;(\mathrm{token}\;\mathrm{projections})\;+\;O\left(BhNd\right)\;(\mathrm{attention}\;\mathrm{core})\;+\;O\left(BNC^{2}\right)\;\left(\mathrm{FFN}\right),$$

with C=hd. Thus, ACT remains linear in token number N=H×W at fixed channel width and head configuration. Relative to the YOLOv11-s baseline, adding CACT keeps the overall giga floating-point operations (GFLOPs) at 21.3 (rounded to one decimal), indicating marginal network-level overhead. Functionally, ACT emphasizes head competition through Π and channel suppression through 1/(1+ψ) while modeling token interactions through token-statistics weighting. Empirical latency/memory scaling of ACT under identical feature resolutions is further reported in Section 5.6.

#### 4.1.7. Second-Stage Modulation to Curb Amplitude Drift (DynamicTanh 2)

Attention redistribution can still cause amplitude drift. We remap to feature space and apply a second DynamicTanh before channel mixing:

(10) $$\begin{array}{cc} U & ={\mathrm{reshape}}^{-1}\left(T^{\prime }\right)+{\mathrm{branch}}_{b}, \end{array}$$

(11) $$\begin{array}{cc} \tilde{T} & ={\mathrm{DYT}}_{2}\left(U\right), \end{array}$$

(12) $$\begin{array}{cc} {\tilde{T}}_{b,c,u,v} & =\gamma_{2,c}\tanh\;\left(\alpha_{2}\cdot U_{b,c,u,v}\right)+\beta_{2,c}, \end{array}$$

where $\alpha_{2}\in R$ and $\gamma_{2},\beta_{2}\in R^{C}$. This rebalances channel magnitudes so the FFN receives well-scaled inputs.

#### 4.1.8. Channel Aggregation with Residual Output (FFN)

To enable stacking multiple ACT blocks without gradient decay, we mix channels via a two-layer feed-forward network (1×1 conv + SiLU + 1×1 conv):

(13) $$\begin{array}{cc} \mathrm{FFN}(\tilde{T}) & =W_{2}\cdot \mathrm{SiLU}\left(W_{1}\cdot \tilde{T}+b_{1}\right)+b_{2},\\ Y & =U+\mathrm{FFN}(\tilde{T}). \end{array}$$

The output $Y\in R^{B\times 256\times H\times W}$ preserves spatial layout while injecting contrastively reweighted semantics with stable gradient flow.

#### 4.1.9. Cumulative Effect and Benefits

Dynamic modulation plus sequencing cleans and enriches features before attention; contrastive attention with second-stage tuning boosts salient regions and corrects spatial bias without runaway amplification; residuals keep gradients stable across stacked ACT blocks. Thus, CACT delivers semantic consistency and spatial alignment for complex weather, providing high-quality priors for classification and regression.

#### 4.1.10. Feature Fusion (Concat + cv2)

The calibrated feature *Y* is concatenated with branch_a and projected back to 512 channels via cv2 (Conv 1×1 + BN + SiLU):

(14) $$\begin{array}{cc} X_{\mathrm{out}} & =\mathrm{cv}2\left(\mathrm{Concat}\left({\mathrm{branch}}_{a},Y\right)\right)\in R^{B\times 512\times H\times W}. \end{array}$$

This serves as input to the next CACT block and, after stacking, feeds the DAAH head alongside ScaleFusion outputs P4/P3 for multi-scale decoding. CACT, therefore, executes a lightweight loop of channel splitting, dynamic alignment, complementary mapping, and feature aggregation, propagating shallow spatial cues into deep semantics and mitigating cross-scale bias for stable detection in adverse weather.

### 4.2. Dynamic Adaptive Alignment Head (DAAH)

**Figure 8:**
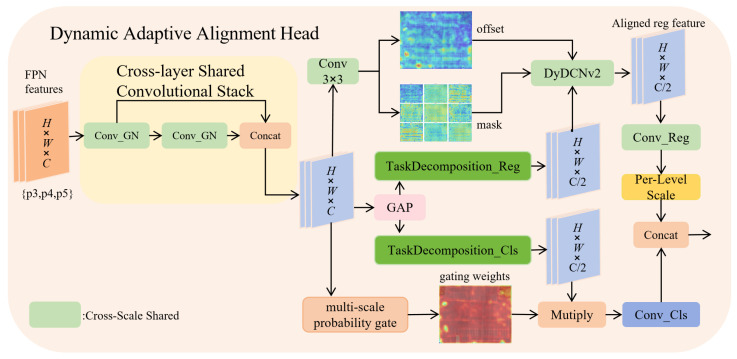
Framework of the Dynamic Adaptive Alignment Head.

Separating classification and regression heads often leads to weak task interaction and inconsistent scales. DAAH addresses this with shared encoding, task decoupling, and dynamic alignment (Figure 8), reducing complexity while jointly optimizing both tasks.

**Shared feature encoding.** Independent feature extractors for each task inflate parameters and memory. DAAH uses shared convolutional layers to fuse shallow and deep features per scale via two conv+GroupNorm (GN) stages, producing

(15) $$\begin{array}{cc} f_{i} & \in R^{B\times C\times H_{i}\times W_{i}}. \end{array}$$

GN stabilizes features, especially for small batches. To handle magnitude differences across scales, a learnable Scale module calibrates each level with a multiplicative gain:

(16) $$\begin{array}{cc} f_{i} & =\alpha_{i}\cdot f_{i}, \end{array}$$

keeping responses aligned and reducing prediction bias.

**Task decoupling with tailored alignment.** Classification focuses on semantics, while regression demands geometric alignment; naive sharing can cause interference. We, therefore, map shared features to task-specific subspaces using global average pooling (GAP) to extract the context, followed by lightweight gating to obtain $f_{i}^{cls}$ and $f_{i}^{reg}$. Context-guided decoupling reduces task interference while preserving shared cues.

Regression alignment relies on deformable convolution (DyDCNv2): predicted offsets $\Delta_{i}$ and masks $m_{i}$ adapt sampling positions and weights, capturing key geometry and improving localization under shape variation and occlusion.

Classification alignment uses multi-scale probabilistic gating. Cascaded 1×1 and 3×3 convolutions produce a gate map,

(17) $$\begin{array}{cc} p_{i} & =\sigma (W_{3\times 3}\cdot \mathrm{ReLU}\left(W_{1\times 1}\cdot f_{i}^{cls}\right)) \end{array}$$

with Sigmoid output in [0,1]. Element-wise modulation, $f_{i}^{cls}=f_{i}^{cls}⊙p_{i}$, enhances informative regions, combining channel interaction and spatial awareness. This suppresses noise and emphasizes high-confidence areas, boosting semantic focus in cluttered or low-light scenes (rain, fog, snow).

**Cross-scale prediction aggregation.** The regression branch maps aligned features to 4 × reg_max channels for distributed predictions (center offsets and size), while the classification branch outputs $n_{c}$ class probabilities. Training keeps all scale outputs $y_{i}$; inference concatenates and decodes via DFL and dist2bbox for final boxes, avoiding redundant multi-scale processing and reducing latency. The Scale module further harmonizes scale responses so weights are distributed consistently across levels.

Overall, DAAH aligns tasks and space on shared encodings: shared convolutions cut parameters, while Scale maintains scale fitness; regression gains geometric adaptivity via DCNv2; and classification focuses semantics via probabilistic gating. This collaborative design improves head stability and reliability under complex weather with minimal overhead.

## 5. Experiments

We validate the proposed approach through comparative and ablation studies.

### 5.1. Implementation Details

All experiments run on an NVIDIA GeForce RTX 4070 Ti GPU (NVIDIA Corporation, Santa Clara, CA, USA) with PyTorch 2.2.2+cu121. We train for 200 epochs with stochastic gradient descent (SGD), momentum 0.937, weight decay 0.0005, batch size 16, and initial learning rate 0.001. Images are resized by letterbox to 640×640; the other training settings follow the official defaults. The evaluation follows COCO-style detection metrics, including AP (AP@[0.50:0.95]), AP_50_, and AP_75_. The inference for evaluation uses single-scale 640×640 inputs with test-time augmentation disabled (augment = False). The predictions are post-processed by non-maximum suppression (NMS) (validation defaults: conf = 0.001, iou = 0.7, max_det = 300), and AP is computed from the post-NMS detections. The baseline in all comparisons is YOLOv11-s (official Ultralytics implementation), trained and evaluated under the same data split, image size, optimizer schedule, and post-processing protocol as DANet; DANet replaces only the designated modules (CACT and/or DAAH) for a controlled comparison.

For speed reporting, we use native PyTorch .pt inference on a single GPU, without TensorRT acceleration. Unless otherwise specified, speed is measured on the SWUAV test split with batch size 16 and single-precision floating point (FP32). We report frames per second, FPS (infer), as $1000/t_{\mathrm{infer}}$ and FPS as $1000/(t_{\mathrm{pre}}+t_{\mathrm{infer}}+t_{\mathrm{post}})$. For YOLOX-s and EdgeYOLO-s, we use external evaluation logs that provide forward and forward+NMS time; therefore, FPS (infer) is derived from forward time and FPS from forward+NMS. To complement the complexity discussion, additional module-level latency/memory performance profiling of ACT is reported in Section 5.6.

### 5.2. Detection Results on SWUAV

We adopt YOLOv11-s as the baseline and compare with UAV baselines of the same input size. As shown in Table 3, YOLOv11-s yields 43.9% AP and 62.6% AP_50_, while our method reaches 46.9% AP and 64.8% AP_50_, improving AP and AP_50_ by 3.0 and 2.2 points. With CACT and DAAH, our network fuses semantics across levels and dynamically aligns classification/regression, markedly boosting detection. The model has 8.65 M parameters and about 22.7 GFLOPs, with 323.47 FPS end-to-end throughput (3.09 ms per image, batch size 16), indicating strong real-time potential.

Table 3 also shows that DANet achieves the highest accuracy among models with the same input size, outperforming RetinaNet (30.4% AP at 640×640) by 16.5 points. Compared with EdgeYOLO-s, DANet attains higher accuracy (46.9% AP) and faster inference throughput (423.82 FPS(infer)) with fewer parameters (8.65 M) and lower computing (22.7 G); its end-to-end speed is 323.47 FPS, demonstrating strong effectiveness for multi-scale detection in UAV imagery. Size-wise AP indicates that gains are more pronounced on medium/large objects (AP_*M*_: 45.2 → 47.1, AP_*L*_: 36.3 → 44.2), while small-object performance remains comparable (AP_*S*_: 14.4 → 14.2).

#### Repeated-Run Stability

To evaluate run-to-run variation, we repeat DANet training and testing three times with different random seeds under the same protocol. As summarized in Table 4, the mean performance is 46.81 AP, 64.89 AP_50_, and 54.77 AP_75_, with low standard deviations (0.12/0.14/0.06). The corresponding 95% confidence intervals are narrow, indicating stable gains across repeated runs, rather than isolated single-run fluctuation.

**Figure 9:**
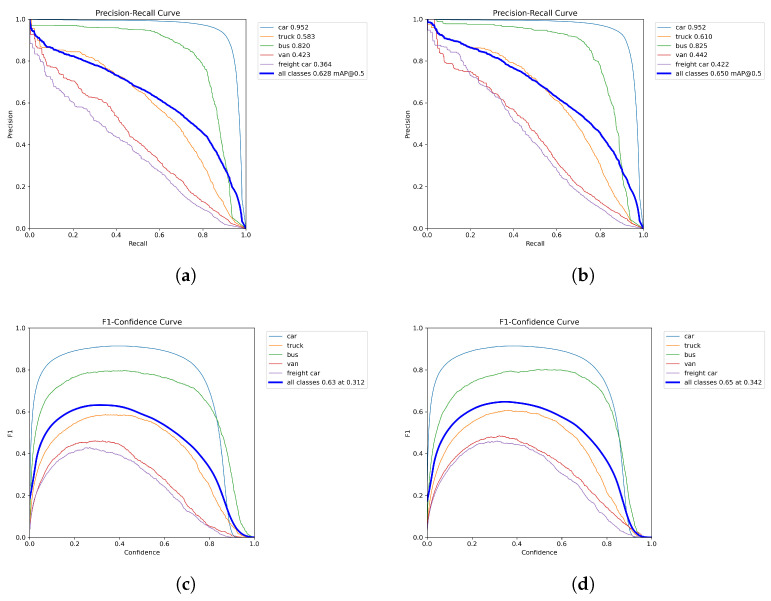
PR and F1-confidence curve comparison between YOLOv11-s baseline and DANet on the WeatherDrone test split. (**a**) YOLOv11-s baseline: PR curve. (**b**) DANet: PR curve. (**c**) YOLOv11-s baseline: F1-confidence curve. (**d**) DANet: F1-confidence curve.

Table 5 reports per-class AP, and Figure 9 further analyzes class-level precision–recall behavior.

Figure 9, together with Table 5, provides a class-level view of the improvement on minority categories. For Truck/Bus/Van/Freight car, AP increases from 40.6/58.3/27.3/25.8 to 43.7/61.0/29.8/30.5. The PR curves show that these classes maintain higher precision over a broad recall range, and the corresponding AP_50_ values in the legends also rise (Truck: 58.3% → 61.0%, Van: 42.3% → 44.2%, Freight car: 36.4% → 42.2%). In the F1-confidence plots, the all-class peak improves from 0.63 to 0.65, with the best operating confidence shifting from 0.312 to 0.342. Under the same evaluation settings, the total number of output detections on the test split is 463,487 for DANet versus 468,735 for YOLOv11-s (about −1.12%). Overall, the curves indicate a more favorable precision-recall balance under severe-weather conditions, especially for low-frequency vehicle classes.

**Figure 10:**
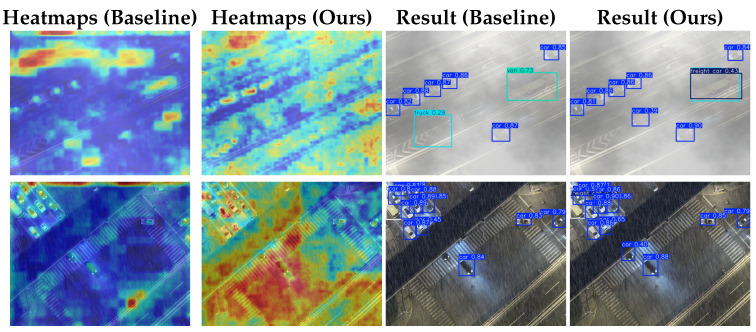
Visualization of heatmaps and detection results for the baseline model and ours.

Figure 10 compares baseline and DANet outputs. Baseline heatmaps focus on misplaced regions and miss targets, indicating strong interference; DANet better adapts to background, covering and analyzing detection areas more completely. In Figure 10, the first-row baseline misdetects a car with a heavily offset box, whereas DANet localizes it correctly and also detects the adjacent truck bed. In the second row, DANet detects a vehicle occluded by an overpass that the baseline misses. DANet thus reduces false detections, yields tighter boxes, and improves detection of medium/large objects and hard occlusion cases in complex low-visibility scenes.

### 5.3. Ablation Experiments

We conduct ablation studies on CACT and DAAH on SWUAV to analyze their impact on accuracy and complexity (Table 6).

The baseline YOLOv11-s achieves 43.9% AP, 62.6% AP_50_, and 51.9% AP_75_ with 9.41 M parameters and 21.3 GFLOPs.

Adding only CACT improves AP to 45.5% (+1.6), AP_50_ by 1.7, and AP_75_ by 1.8 while slightly reducing parameters to 9.35 M with a negligible computational overhead. This indicates that cross-scale adaptive contrastive calibration enhances feature consistency across different semantic levels under adverse conditions without increasing model complexity.

Using only DAAH raises AP to 46.4% (+2.5), with the largest gain observed in AP_75_ (+2.7), suggesting that deformable alignment and probabilistic gating effectively improve localization and classification quality, particularly under stricter intersection over union (IoU) thresholds. The parameters are reduced to 8.71 M, while GFLOPs increase to 22.7 due to the use of deformable convolution.

Enabling both modules yields 46.9/64.8/54.4 for AP/AP_50_/AP_75_ (gains of 3.0/2.2/2.5) while compressing parameters to 8.65 M with a slight increase in computation. Relative to CACT-only, adding DAAH brings +1.4/+0.5/+0.7 AP/AP_50_/AP_75_; relative to DAAH-only, adding CACT brings +0.5/+0.2/−0.2. This indicates that CACT and DAAH are partially complementary with overlapping effects.

### 5.4. Cross-Dataset Experiments

To evaluate cross-dataset generalization, we directly test models trained on SWUAV on VisDrone-DET and RTTS without target-domain training. VisDrone uses the vehicle-only val split (car, truck, bus, van) and retains negative images; RTTS keeps only the overlapping car and truck classes and retains negative images without car/truck to keep evaluation strict. Table 7 reports target-domain metrics together with source-to-target degradation from SWUAV, and Table 8 provides a per-category AP_50_ degradation breakdown.

Cross-dataset evaluation indicates substantial source-to-target degradation from SWUAV to both VisDrone and RTTS. In AP_50_, YOLOv11-s drops by 51.84 and 59.81 percentage points on VisDrone and RTTS, while DANet (Ours) drops by 53.75 and 61.93, respectively, confirming severe domain shift for both models.

The per-category AP_50_ analysis shows that degradation is strongly class-dependent. On VisDrone, the bus exhibits the largest drop (80.30/81.14 p.p. for YOLOv11-s/DANet), while the car and truck are comparable in magnitude (61.02/58.81 and 57.62/60.38 p.p.), and the van is relatively smaller (34.91/37.53 p.p.). On RTTS, both shared classes are heavily degraded, with the car at around 89.5 p.p. for both models. These patterns indicate that cross-domain degradation is heterogeneous across categories, rather than uniform.

Under strictly controlled settings (identical SWUAV source training data, detector family, and evaluation protocol), DANet (Ours) still yields consistent but modest target-domain gains in AP_50_/AP_75_/AP and F1 on both datasets (VisDrone: +0.29/+0.21/+0.16 AP_50_/AP_75_/AP points; RTTS: +0.08/+0.11/+0.11). Although improvements are more stable on dominant categories, gains are not exclusively limited to a single class. In addition, DANet (Ours) shows a higher target recall but slightly lower target precision, indicating a recall-oriented trade-off, rather than indiscriminate over-prediction. Taken together, these observations suggest that the gains mainly arise from improved architectural robustness/generalization, while the residual dataset mismatch remains.

### 5.5. Internal Ablation of DAAH

To further clarify the role of each alignment component under an unchanged framework, we perform a component-wise internal ablation of DAAH on the SWUAV validation split, as summarized in Table 9. The first row reports the CACT-enhanced model with the native YOLOv11 detection head (without DAAH), while the remaining rows report DAAH variants with different combinations of DyDCNv2, probabilistic gating, and scale calibration.

Compared with the CACT model using the native head, replacing the head with a DAAH variant while disabling all internal alignment components yields only limited gains. On top of this fixed DAAH structure, enabling alignment components further increases AP/AP_50_/AP_75_ from 46.20/64.31/54.44 to 46.97/65.17/55.33; this alignment-induced improvement accounts for 51.3%, 78.2%, and 73.0% of the total gains over the CACT model with the native head, respectively, indicating that the main performance improvement comes from the alignment mechanism itself, especially under stricter IoU criteria. Component-wise, removing DyDCNv2 causes the largest degradation (46.97 → 45.50 AP), and removing probabilistic gating also reduces performance (46.97 → 46.58 AP), showing that geometric adaptive sampling and semantic gating are the primary drivers. In contrast, scale calibration has a more moderate effect on AP but still improves AP_50_, AP_75_, and F1 calibration in the full configuration. Overall, DyDCNv2, probabilistic gating, and scale calibration act complementarily, with DyDCNv2 and gating playing the dominant roles.

### 5.6. Latency and Memory Scaling Analysis of ACT

To validate ACT efficiency in practice, we perform module-level performance profiling against a comparison module, i.e., C2PSA in YOLOv11, which uses standard global self-attention. Unless otherwise specified, the performance profiling follows the setup in Section 5.1. The module-specific settings are: $c_{1}\;=\;c_{2}\;=\;1024$, n=2, e=0.5, FP32, batch size 1, and forward-only evaluation. The inputs are $R^{1\times 1024\times S\times S}$ with S∈{20,40,60,80,96,112,128,144}. For each resolution, we use 10 warmup interations and 40 timed iterations (CUDA events). The memory is reported as incremental peak allocation, defined as $\Delta M=M_{\mathrm{peak}}-M_{\mathrm{base}}$, where $M_{\mathrm{peak}}$ denotes the peak allocated GPU memory during timed forward passes, and $M_{\mathrm{base}}$ denotes the allocated GPU memory immediately before timing.

At 20×20 (N=400), standard attention can be faster because fixed runtime overhead dominates. From 40×40 onward, ACT is consistently faster, and the latency gap widens with resolution. At 144×144, standard attention reaches OOM, while ACT still runs. Local non-monotonic fluctuations of ΔM (e.g., 80×80 vs. 96×96 for ACT) are expected from CUDA allocator reuse and shape-dependent backend workspace selection; however, the overall trend and high-resolution boundary remain consistent with the linear-token complexity analysis. The detailed resolution-wise latency and memory results are summarized in Table 10.

## 6. Conclusions

We present SWUAV, a new severe-weather UAV aerial dataset with 18,195 images across 12 adverse weather and illumination conditions and diverse traffic scenes, providing a challenging basis for UAV traffic detection in complex environments. We also propose DANet, a detection framework that improves robustness under adverse-weather conditions, combining the Cross-Scale Adaptive Contrastive Tokenizer (CACT) and the Dynamic Adaptive Alignment Head (DAAH). CACT aligns shallow geometric details with deep semantics in high-resolution space via channel splitting and contrastive attention, alleviating semantic fading under adverse-weather noise, especially for hard and occluded targets; DAAH introduces deformable convolution and probabilistic gating on shared encodings to dynamically align classification/regression and multi-scale features, improving stability and discrimination with controlled parameters and computing. DANet improves accuracy and enhances robustness in complex real-world conditions, including low visibility, heavy occlusion, and mixed vehicle types. Within this scope, the image-formation terms are used as a practical mixed-degradation representation to support improved detection performance under diverse adverse weather and illumination conditions.

Extensive experiments on SWUAV show that DANet surpasses mainstream detectors on AP, AP_50_, and AP_75_, especially in dense traffic and adverse weather. Ablations confirm the effectiveness of CACT and DAAH, particularly for multi-scale alignment and minority-class recognition. Future work will extend SWUAV and DANet to more dynamic traffic and UAV trajectory scenarios, and incorporate multimodal sensors (e.g., infrared, thermal) to further boost detection under harsh weather. Overall, SWUAV and DANet demonstrate improved performance and generalization under diverse conditions and provide a strong data/tooling foundation for traffic detection in severe-weather UAV imagery.

## Figures and Tables

**Table 1:** Weather and illumination conditions covered by SWUAV.

Weather/Illumination	Brief Description
Heavy Rain	Downpour, spray, wet glare.
Snowfall/Blizzard	Drifting flakes, obscured markings.
Freezing Rain/Sleet	Ice pellets, wiper occlusion.
Dense Fog	Visibility < 100 m, halo effects.
Haze/Smog	Low contrast, color shift.
Mist/Light Drizzle	Fine droplets, mild highlights.
Clear Night	Streetlamp lighting, deep shadows.
Backlit Night	Headlight/neon glare.
Dusk/Dawn	Low sun, warm tint.
Overcast Day	Diffused soft light.
Harsh Sunlight	High contrast, highlights.
Emergency Lighting	Flashing patrol/rescue beacons.

**Table 2:** Weather distribution comparison between SWUAV and UAVDT-M.

Dataset	Weather	Images	Percentage
SWUAV	Heavy Rain	3468	19.06%
SWUAV	Snowfall/Blizzard	1542	8.47%
SWUAV	Freezing Rain/Sleet	2104	11.56%
SWUAV	Dense Fog	3016	16.58%
SWUAV	Haze/Smog	1226	6.74%
SWUAV	Mist/Light Drizzle	2689	14.78%
SWUAV	Clear Night	1062	5.84%
SWUAV	Backlit Night	812	4.46%
SWUAV	Dusk/Dawn	336	1.85%
SWUAV	Overcast Day	1480	8.14%
SWUAV	Harsh Sunlight	350	1.92%
SWUAV	Emergency Lighting	110	0.60%
SWUAV	Total	18,195	100%
UAVDT-M	Daylight	24,055	59.05%
UAVDT-M	Night	11,501	28.23%
UAVDT-M	Fog	5179	12.71%
UAVDT-M	Total	40,735	100%

**Table 3:** Comparison with state-of-the-art detectors on the SWUAV test set in terms of accuracy, computing, parameters, and speed. FPS(infer) denotes forward throughput, while FPS denotes end-to-end throughput (preprocess + inference + postprocess). The full speed protocol is described in Section 5.1.

Model	AP (%)	AP_50_ (%)	AP_75_ (%)	AP_*S*_ (%)	AP_*M*_ (%)	AP_*L*_ (%)	GFLOPs	Params	FPS (Infer)	FPS
EfficientDet-D2 [16]	34.8	48.1	38.8	/	/	/	7.6 G	8.1 M	128.70	/
PPYOLOE-s [29]	35.5	48.3	40.5	/	/	/	17.3 G	7.9 M	158.00	/
RetinaNet-R50 [30]	30.4	42.9	35.6	/	/	/	/	/	/	/
Cascade R-CNN-R50 [31]	39.5	55.5	47.0	/	/	/	/	/	/	/
Faster R-CNN-R50 [12]	38.5	53.1	45.8	/	/	/	/	/	/	/
YOLOX-s [32]	42.6	61.2	50.2	/	/	/	26.77 G	8.94 M	493.58	370.87
EdgeYOLO-s [33]	41.6	64.1	47.8	/	/	/	45.3 G	9.9 M	145.35	132.28
CGMDet [34]	46.0	64.6	/	/	/	/	25.51 G	5.59 M	263.00	/
RT-DETR-R18 [35]	39.6	54.7	46.1	11.4	41.3	30.5	77.8 G	25.19 M	172.49	163.75
RT-DETR-R34 [35]	40.4	56.1	46.9	11.2	42.1	32.1	96.3 G	31.37 M	146.78	140.47
YOLOv5-s [36]	42.4	60.2	50.0	12.8	43.8	35.4	18.7 G	7.82 M	614.57	423.56
YOLOv8-s [37]	45.7	64.5	54.0	13.8	47.1	38.8	23.4 G	9.83 M	306.45	226.39
YOLOv9-s [38]	44.8	63.5	53.1	14.1	45.2	39.7	22.1 G	6.20 M	252.75	189.34
YOLOv10-s [39]	41.6	59.3	49.2	14.0	43.0	33.4	21.4 G	7.22 M	269.21	248.13
YOLOv11-s [40]	43.9	62.6	51.9	14.4	45.2	36.3	21.3 G	9.41 M	539.03	382.06
YOLOv12-s [41]	44.9	64.1	53.3	13.8	46.1	40.2	19.3 G	9.08 M	297.68	215.65
DANet (Ours)	46.9	64.8	54.4	14.2	47.1	44.2	22.7 G	8.65 M	423.82	323.47

**Table 4:** Repeated-run stability of DANet on the SWUAV test split (three seeds).

Run	AP	AP_50_	AP_75_
seed1	46.84	64.88	54.78
seed2	46.68	64.76	54.71
seed3	46.91	65.04	54.83
Mean ± Std	46.81 ± 0.12	64.89 ± 0.14	54.77 ± 0.06
95% CI	[46.52, 47.10]	[64.54, 65.24]	[54.62, 54.92]

**Table 5:** Per-class AP on the WeatherDrone test split.

Model	AP	Car	Truck	Bus	Van	Freight Car
YOLOv11-s (baseline)	44.2	69.3	40.6	58.3	27.3	25.8
DANet (Ours)	46.9	69.5	43.7	61.0	29.8	30.5

**Table 6:** Ablation of CACT and DAAH in DANet on the test set. Checkmark denotes enabled and blank denotes disabled.

CACT	DAAH	AP	AP_50_	AP_75_	Params (M)	GFLOPs
		43.9	62.6	51.9	9.41	21.3
✓		45.5	64.3	53.7	9.35	21.3
	✓	46.4	64.6	54.6	8.71	22.7
✓	✓	46.9	64.8	54.4	8.65	22.7

**Table 7:** Cross-dataset evaluation and source-to-target degradation from SWUAV.

Model	Test Set	Precision (%)	Recall (%)	F1 (%)	AP_50_ (%)	AP_75_ (%)	AP (%)	AP_50_ Drop (p.p.)	AP Drop (p.p.)	Recall Drop (p.p.)
YOLOv11-s	VisDrone	19.87	14.50	14.48	11.00	4.66	5.45	51.84	38.78	48.40
DANet (Ours)	VisDrone	18.39	16.18	14.98	11.29	4.87	5.61	53.75	41.30	46.89
YOLOv11-s	RTTS	11.17	5.96	6.74	3.03	0.61	1.07	59.81	43.16	56.94
DANet (Ours)	RTTS	10.34	7.50	7.47	3.11	0.72	1.18	61.93	45.73	55.57

**Table 8:** Per-category AP_50_ source-to-target degradation (shared classes only).

Target Test Set	Class	YOLOv11-s Drop (p.p.)	DANet (Ours) Drop (p.p.)	YOLOv11-s Target AP_50_ (%)	DANet (Ours) Target AP_50_ (%)	Target Gain (p.p.)
VisDrone	car	61.02	58.81	34.18	36.43	2.25
VisDrone	bus	80.30	81.14	1.70	1.36	−0.34
VisDrone	truck	57.62	60.38	0.72	0.65	−0.07
VisDrone	van	34.91	37.53	7.39	6.71	−0.68
RTTS	car	89.47	89.44	5.73	5.80	0.07
RTTS	truck	58.01	60.61	0.33	0.42	0.09

**Table 9:** Internal ablation of DAAH on SWUAV validation split. Checkmark denotes enabled and blank denotes disabled.

DAAH	DyDCNv2	Gating	Scale	AP	AP_50_	AP_75_	Recall	F1
	–	–	–	45.47	64.07	54.11	62.95	63.90
✓				46.20	64.31	54.44	63.03	64.48
✓	✓			45.82	64.01	53.76	62.91	63.14
✓		✓		46.72	64.94	55.13	63.50	64.72
✓			✓	45.98	64.08	54.75	63.78	63.42
✓		✓	✓	45.50	63.89	53.28	63.19	63.92
✓	✓		✓	46.58	64.72	54.67	63.02	64.03
✓	✓	✓		46.98	64.88	55.09	63.12	63.93
✓	✓	✓	✓	46.97	65.17	55.33	62.88	65.00

**Table 10:** Module-level latency and memory scaling of ACT versus a standard self-attention baseline under identical feature resolutions.

Resolution	ACT Latency (ms)	ACT ΔM (MB)	Standard SA Latency (ms)	Standard SA ΔM (MB)
20×20	2.1987	132.78	1.0942	131.12
40×40	1.5740	22.80	2.8917	165.62
60×60	2.1142	170.19	11.7196	813.09
80×80	3.7539	190.50	34.5918	2537.50
96×96	5.4031	126.29	68.7199	5238.00
112×112	7.7243	175.50	210.5959	9678.50
128×128	13.1860	224.50	6117.7465	16,480.00
144×144	14.8115	287.50	OOM	OOM

## Data Availability

The SWUAV dataset, including raw UAV images and annotations, is available at https://github.com/Chien-Abyss/SWUAV-DANet (accessed on 3 February 2026). The source code of the proposed Dynamic AlignAir Network is openly available at the same repository.
